# Multiple bile duct adenomas mimicking hepatic metastases during laparoscopic cholecystectomy - A case report

**DOI:** 10.1016/j.ijscr.2025.111082

**Published:** 2025-02-21

**Authors:** Duminda Subasinghe, Jesuthasan Mithushan, Anjana Abeysinghe, Rachini Withanage, Gayani Ranaweera

**Affiliations:** a Department of Surgery, Faculty of Medicine, University of Colombo, Sri Lanka; b University Surgical Unit, The National Hospital of Sri Lanka, Colombo, Sri Lanka; c Department of Anesthesiology and Critical Care, Faculty of Medicine, University of Colombo, Sri Lanka; d Department of Pathology, Faculty of Medicine, University of Colombo, Sri Lanka

**Keywords:** Bile duct adenomas, Laparoscopic cholecystectomy, Hepatic metastases

## Abstract

**Introduction and importance:**

Bile duct adenoma (BDA) is a rare benign liver neoplasm, with relatively few cases reported in literature.

**Case presentation:**

A 55-year-old man diagnosed with symptomatic gallstone disease who underwent elective laparoscopic cholecystectomy. During the procedure, there were multiple liver nodules mimicking hepatic metastases. Histological examination and contrast-enhanced computed tomography (CECT) of the liver confirmed the diagnosis of BDA.

**Discussion:**

When incidental liver nodules are encountered intraoperatively, thorough evaluation is essential to confirm the diagnosis. Structured approach using histological examination and imaging (CECT of the liver/ MRI) confirmed the diagnosis of BDA.

**Conclusions:**

BDA is a rare benign liver neoplasm that can be challenging to differentiate from hepatocellular carcinoma or liver metastases. Accurate identification of BDA can prevent overtreatment or mismanagement.

## Highlights

• Rare Clinical Presentation: The manuscript discusses a rare case of multiple bile duct adenomas (BDAs), a benign condition, which [[1], [2], [3], [4], [5], [6], [7], [8], [9], [10], [11], [12], [13], [14],^13^,^14^] mimicked malignant hepatic metastases, presenting a diagnostic and therapeutic challenge.
• Significance in Differential Diagnosis: Emphasizes the importance of considering BDAs in the differential diagnosis of liver lesions observed intraoperatively.
• Role of Histopathology: Highlights importance of histopathological evaluation were critical in confirming the benign nature of the lesions, thus avoiding unnecessary aggressive surgical interventions.
• Imaging and Diagnostic Challenges: Discusses the limitations of preoperative imaging in differentiating BDAs from metastatic disease, stressing the need for intraoperative decision-making based on direct visualization and biopsy.
• Clinical Implications and Outcomes: Provides insights into surgical decision-making, emphasizing minimally invasive management strategies and the importance of avoiding overtreatment in cases of suspected metastatic liver lesions with benign pathology.

## 1 Introduction

Bile duct adenoma (BDA) is a rare, benign tumor, most often discovered incidentally during laparoscopic surgeries, cross sectional imaging or autopsies [1]. According to Craig et al., bile duct adenoma was found in only five out of 50,000 autopsies [2]. It occurs across all age groups and both sexes, showing no gender predilection. Clinically, BDA is typically asymptomatic and exhibits variable detectability through cross-sectional imaging modalities [1,3]. An ultrasound scan (USS) may depict BDA as a hyperechoic lesion with a hypoechoic rim, though it is often not clearly visualized [4]. In contrast, on contrast-enhanced CT (liver protocol) and Gd-enhanced MRI, BDAs typically appear hyper vascular with arterial-phase and delayed-phase enhancement. The persistent enhancement on dynamic imaging may reflect the presence of fibrous stroma within the tumor [4]. The role of Diffusion-weighted magnetic resonance imaging (DWI-MRI) in evaluating BDA is still emerging. Currently, no specific biomarkers are available for their preoperative identification [5]. This makes it challenging to accurately determine its true incidence.

BDA typically presents as a solitary, subcapsular white or grey nodule measuring 1–2 cm in diameter, composed of proliferating small, bland bile ductules. Histopathologically, it is characterized by unencapsulated nodules with well-defined margins, proliferative uniform ductules embedded in fibrotic connective tissue, and the absence of atypia or mitotic activity [1,6].

Anyhow traditional histopathology alone is often insufficient when immunohistochemical analysis is needed to differentiate bile duct adenoma (BDA), particularly in cases associated with pancreatic or hepatocellular carcinoma. Diagnostically challenging BDAs can be distinguished from metastatic pancreatic adenocarcinoma using albumin bISH (Branched-chain in-situ hybridization) [7]. Immunohistochemical markers such as CD10, CK19, CK7, and CD56 are typically positive in BDA, whereas AFP and p53 are usually negative [8].

While the exact etiology of BDA remains uncertain, it is widely considered a reactive process to localized injury rather than a true neoplasm [9]. Nevertheless, some cases of BDA have shown progression to cholangiocarcinoma or coexistence with it [10]. The 5th Edition of the World Health Organization (WHO) Classification of Digestive System Tumors highlights the need for further evaluation of such lesions [11]. Key differential diagnoses for intraoperative solitary lesions include von Meyenburg complexes (bile duct hamartomas), cholangiocarcinoma, and metastatic carcinoma.

This report presents a case of incidental multiple liver nodules identified during laparoscopic cholecystectomy, subsequently confirmed as bile duct adenoma through histopathological evaluation. This case report has been reported in line with SCARE guidelines 2023 [12].

## 2 Case presentation

A 55-year-old ASA I male presented with a single episode of biliary colic lasting two weeks. He had no prior symptoms suggestive of cholangitis, and his past medical and surgical history was unremarkable. Physical examination, including abdominal assessment, revealed no abnormalities. Routine laboratory investigations, including liver function tests and a complete blood count, were within normal limits. His ultrasound abdomen showed multiple gallbladder calculi and there were no liver lesions. He was scheduled for an elective laparoscopic cholecystectomy.

**Fig. 1:**
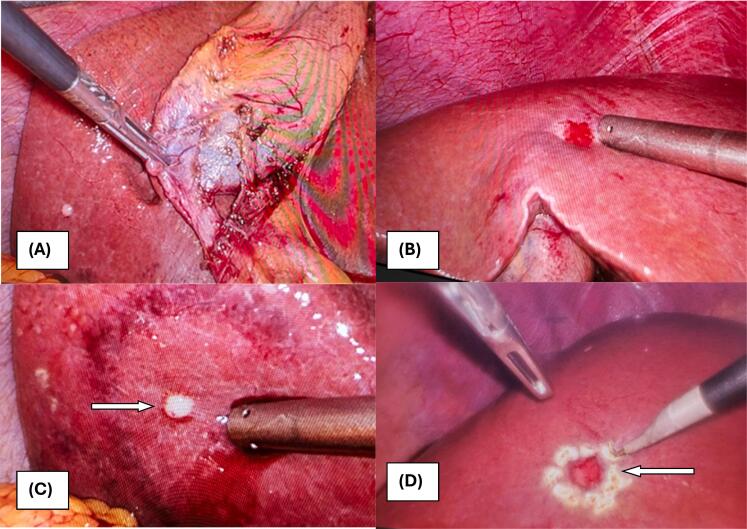
Intraoperative findings of bile duct adenomas. (A) Segment VI lesions and Critical view of safety during laparoscopic cholecystectomy, (B) Multiple whitish liver lesions in segment VI, (C) Nodular liver lesion on segment IV B, (D) Laparoscopic wedge resection of segment IV B liver lesion.

Intraoperatively, multiple firm to hard, whitish nodules were identified on liver segments II, IVB, VI, and VIII (Fig. 1). There were multiple gallbladder calculi without evidence of acute cholecystitis. The rest of the peritoneal cavity appeared normal, without evidence of ascites or other abnormalities. A laparoscopic cholecystectomy was performed with a clear demonstration of the critical view of safety. Additionally, laparoscopic wedge biopsies of liver lesions from segments IVB and VI were obtained for histopathological analysis. The patient's postoperative recovery was uneventful.

**Fig. 2:**
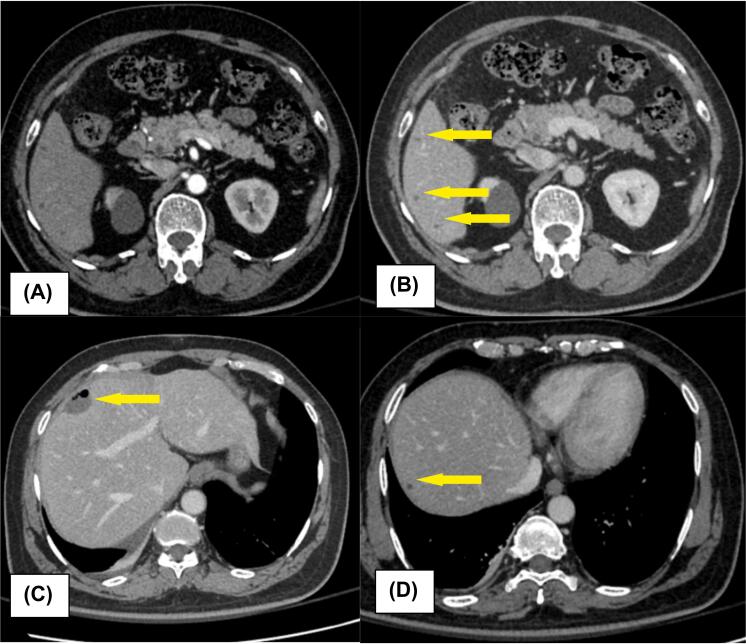
CECT liver scan findings (A) Axial sections of arterial phase, (B) Axial sections of CT (Venous phase) showing multiple bile duct adenomas(BDA) in segment VI, (C) Laparoscopic wedge biopsy site with post-surgical changes, (D) Segment VIII BDA.

**Fig. 3:**
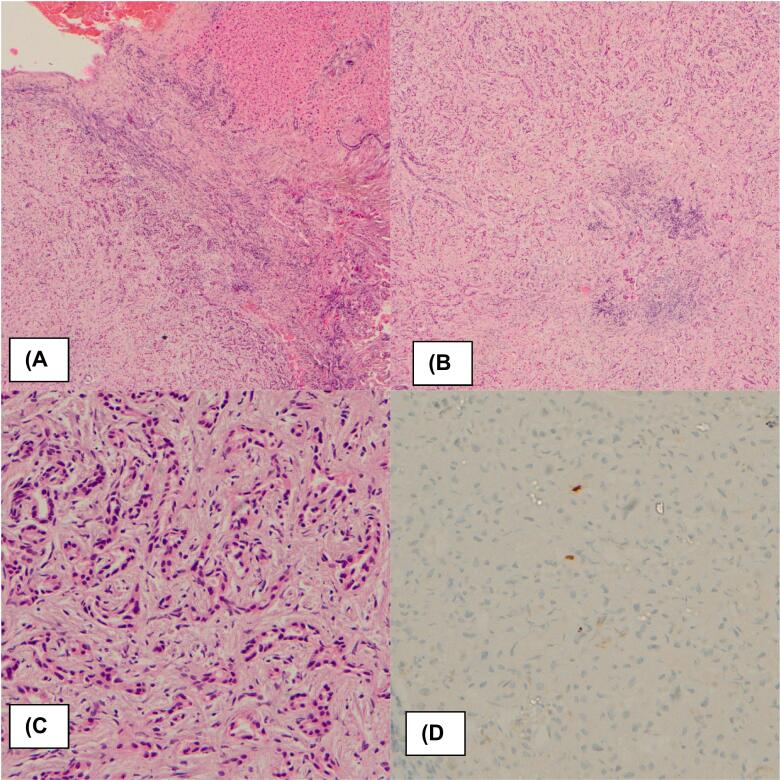
Histopathological appearance of hepatic bile duct adenoma. (A) Well defined lesion with adjacent hepatic parenchymal tissue (H&E × 40); (B) Disorderly arranged, small ducts within a collagenous stroma (H&E × 40); (C) Disorderly arranged, small ducts within a collagenous stroma (H&E × 400); (D) Ki 67 stain - low proliferative index (×400).

Two weeks postoperatively, a CECT liver scan revealed a well-defined cystic lesion (2.2 × 1.8 cm) in the subcapsular region of liver segment VIII, along with multiple smaller lesions in the right lobe and segment II, suggestive of nonspecific liver cysts (Fig. 2). Histopathological examination of liver biopsies revealed a well-defined, non-infiltrative lesion characterized by disorderly arranged, uniform small ducts embedded within a collagenous stroma, interspersed with scattered lymphocytes and plasma cells. The ducts were lined by cuboidal cells with bland nuclei, without evidence of cellular atypia or mitotic activity. Immunohistochemical analysis showed a Ki-67 proliferation index of less than 2 %. These findings were consistent with the bile duct adenoma (Fig. 3). Further evaluation with colonoscopy showed normal colonic mucosa up to the cecum. Tumor markers, including CA 19–9, CEA, and AFP were within normal limits. A repeat full liver function test and follow-up imaging at three months post-surgery showed no abnormalities.

Given that BDA is a benign lesion, and the patient had an uneventful postoperative recovery, routine long-term follow-up was not deemed necessary. However, the patient was advised to seek medical attention promptly if any clinical symptoms arise.

## 3 Discussion

Bile duct adenoma (BDA) is a rare benign hepatic lesion, often identified incidentally during abdominal surgeries or cross-sectional imaging. The clinical presentation of BDA, as in this case, is generally asymptomatic, with the condition usually diagnosed postoperatively based on histopathological findings. In patients with diagnosed adenocarcinoma of the gastrointestinal tract, it can also be falsely identified as liver metastasis [3]. In cirrhotic patients undergoing routine surveillance, BDA can sometimes be misinterpreted as hepatocellular carcinoma [8]. This report highlights the importance of recognizing BDA as a potential diagnosis for liver nodules detected intraoperatively, especially given its ability to mimic more serious pathologies like hepatocellular carcinoma and liver metastases.

Preoperative abdominal ultrasound (USS), contrast-enhanced CT (CECT), and MRI (T1- and T2-weighted imaging) demonstrate variable sensitivity and specificity for detecting BDA [14]. USS may show BDA as a hyperechoic lesion with a hypoechoic rim, though it is often not clearly visualized. In our patient, preoperative abdominal USS was reported as normal, likely due to the small size of the nodule, the limited sensitivity of USS, and its operator-dependent nature. In contrast, contrast-enhanced CT (liver protocol) and Gd-enhanced MRI typically reveal BDAs as hypervascular lesions with arterial-phase and delayed-phase enhancement. However, aside from USS, routine cross-sectional imaging is generally not performed in patients with symptomatic gallstone disease unless there is clinical suspicion of malignancy, as this approach is guided by cost-effectiveness and practicality. The role of diffusion-weighted MRI (DWI-MRI) in evaluating BDA remains under investigation. Consequently, liver nodules in such patients are often detected incidentally during surgery.

In this patient, multiple whitish liver nodules were identified during laparoscopic cholecystectomy, raising concerns for malignancy or other pathologies. In that case, it is essential to perform histopathological evaluation and advanced imaging studies to assess the potential for malignancy.

In our setting, post operative contrast-enhanced computed tomography (CECT) is the commonly utilized imaging modality due to its widespread availability compared to MRI. However, few literatures suggest that magnetic resonance imaging (MRI) of the liver may provide diagnostic accuracy for these lesions [3]. In our patient, postoperative contrast-enhanced CT (CECT) of the liver revealed a cystic lesion in segment VIII along with smaller nonspecific cysts in the right lobe, consistent with the benign nature of BDA. However, as reported in the literature, the characteristic hypervascular appearance with arterial-phase and delayed-phase enhancement was not clearly evident. This highlights the value of integrating multiple imaging modalities with histological assessment for the accurate diagnosis of BDA.

In our patient, the subsequent histopathological findings of unencapsulated nodules with uniform bile ductules in fibrotic stroma confirmed the diagnosis of BDA. This enables us to exclude other differential diagnosis such as, von Meyenburg complexes, focal nodular hyperplasia, cholangiocarcinoma, and metastatic carcinoma. Further, immunohistochemical analysis differentiates BDA by its low ki67 index less than 2%, negative p53 stains a feature that contrasts with malignancy. Although advanced immunohistochemical markers such as albumin, CD10, CK19, CK7, and CD56 offer greater diagnostic accuracy, their limited availability and high cost in resource-constrained settings may hinder the diagnosis of complex cases.

Additional investigations, including tumor markers to assess liver metastases and lower endoscopy to identify potential primary lower gastrointestinal malignancies, were conducted. These supplementary tests further corroborated the diagnosis of BDA.

Although BDA is classified as a benign neoplasm, reports suggest it carries a potential risk of malignant transformation into cholangiocarcinoma [14]. Consequently, surgical resection or local excision is recommended, as these interventions yield favorable outcomes. Notably, no cases of tumor recurrence following resection have been documented in the literature to date [13]. However, surgical resection approaches, surveillance protocols, and postoperative follow-up strategies remain largely uncharted and require further exploration.

This case underscores the clinical importance of a systematic approach to incidental liver lesions. Careful intraoperative assessment, combined with targeted biopsies and subsequent histopathological analysis, is essential for accurate diagnosis and appropriate management. In this patient, the incidental identification of BDA had no immediate clinical implications, and the patient's postoperative course was uneventful. Nevertheless, the case highlights the significance of distinguishing BDA from other hepatic pathologies to avoid overtreatment or mismanagement.

## 4 Conclusions

BDA remains a rare but important differential diagnosis for incidental hepatic nodules. A multidisciplinary approach involving surgical, radiological, and pathological expertise is crucial to ensuring accurate diagnosis and optimal patient outcomes. Further studies on BDA are warranted to better understand its pathogenesis, potential for malignancy, and long-term clinical implications.

## Informed consent

An informed written consent was taken from the patient for publication and any accompanying images. A copy of the written consent is available for review by the Editor-in-Chief of this journal on request.

## Ethical approval

NHSL Ethics review committee: Ethical approval was deemed unnecessary by the institutional ethics committee as the paper reports a single case that emerged during normal practice.

## Guarantor

Duminda Subasinghe.

## Declaration of Generative AI and AI-assisted technologies in the writing process

AI was utilized exclusively to refine language, enhance clarity, and improve readability in this manuscript. No substantive content was generated, modified, or altered by AI.

## Funding

This research received no specific grant from any funding agency in the public, commercial, or not-for-profit sectors.

## CRediT authorship contribution statement

**Duminda Subasinghe:** Writing – review & editing, Supervision. **Jesuthasan Mithushan:** Writing – original draft, Writing – review & editing. **Anjana Abeysinghe:** Writing – review & editing. **Rachini Withanage:** Writing – review & editing. **Gayani Ranaweera:** Writing – review & editing.

## Declaration of competing interest

The Author(s) declares(s) that there is no conflict of interest.
